# Left Anterior Descending Artery Bridging and Atypical Chest Pain in a Young Woman

**DOI:** 10.7759/cureus.10550

**Published:** 2020-09-20

**Authors:** Mohanad Ahmed, Khalid Hassan, Mohamed Elfatih Mustafa

**Affiliations:** 1 Internal Medicine, Hamad Medical Corporation, Doha, QAT; 2 Medicine, Hamad Medical Corporation, Doha, QAT

**Keywords:** chest pain, lad bridge, ischemic heart disease, myocardial bridge

## Abstract

Cardiac-related chest pain is a frequent cause of morbidity and mortality and should be carefully assessed due to its burden on patient health. Its etiology can sometimes prove challenging to discover because of atypical presentations or rare causes of chest pain like myocardial bridging (MB). MB requires a high index of suspicion to be diagnosed. MB is a rare congenital anomaly that occurs due to the passage of a segment of a coronary artery inside the myocardium, causing chest pain due to compression during systole. MB usually has no clinical significance in most cases. However, when severe bridging occurs in the major coronary arteries, patients can experience myocardial ischemia, coronary thrombosis, myocardial infarction, and stress cardiomyopathy, leading to arrhythmias and sudden death. We present the case of a young woman who presented with atypical (rather than ischemic) chest pain due to MB.

## Introduction

Myocardial bridging (MB) occurs when an epicardial segment of the coronary artery [usually the left anterior descending (LAD) artery] goes intramyocardially, causing chest pain due to systolic compression [1]. MB was first discovered at an autopsy in 1737 and was first described by angiography by Porstmann et al. [2]. MB was first managed with myotomy by Binet et al. [3]. The condition affects men more than women (2:1 ratio) and manifests clinically at 40-50 years of age [2,4,5]. Symptoms range from a typical chest discomfort to an atypical chest pain inconstantly related to physical or psychic stress [2,4,5]. The treatment for MB involves medications like beta-blockers and calcium channel blockers or surgery if medications fail. We present a rare case of mid-LAD bridging, which had resulted in recurrent atypical (rather than ischemic) chest pain in a young woman.

## Case presentation

A 24-year-old woman presented to our clinic with a recurrent stabbing pain on her left side, below the nipple. She reported the pain as 8/10 in intensity. She noted that her pain was intermittent and sometimes occurred at rest, too. She had no associated shortness of breath, but the pain was associated with mild palpitation. Her symptoms were such that she had presented to the emergency department many times, and she had been diagnosed with muscular pain at each visit and discharged on simple analgesics. The patient was known to have hypothyroidism on replacement therapy with 50 mcg of levothyroxine, and she had a history of mild iron deficiency anemia due to heavy menses and an iron replacement regimen. She was not a smoker and did not consume alcohol, nor had she had any previous surgeries.

On assessment, her vital signs were within reference limits, and the findings of her systemic examination were unremarkable. Her laboratory investigations results were also within reference limits, including cardiac enzymes. Her hemoglobin was 10.5 mg/dl, ferritin was 11 µg/L, and her glycated hemoglobin concentration was 5.3%. Her renal function, electrolytes, thyroid function, and liver function were all within reference limits.

**Figure 1:**
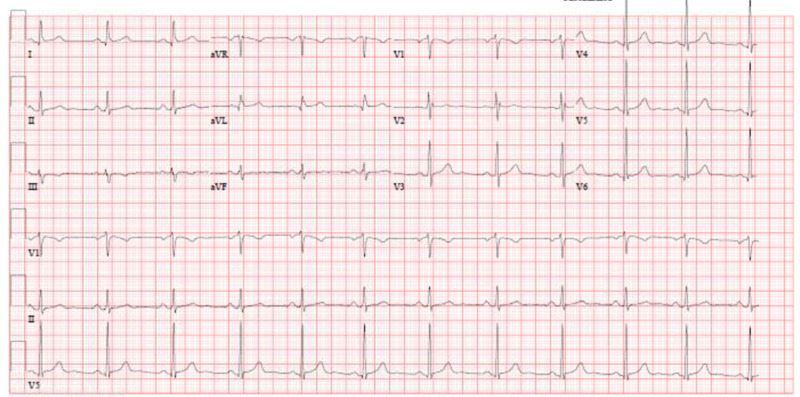
ECG at rest showed normal sinus rhythm ECG: electrocardiogram

We conducted an electrocardiogram (Figure 1), chest X-ray, and echocardiography, the results of which were all within reference limits and/or unremarkable. Her heart rate response to exercise was appropriate, and her blood pressure was within reference range at rest but blunted in response to exercise.

**Figure 2:**
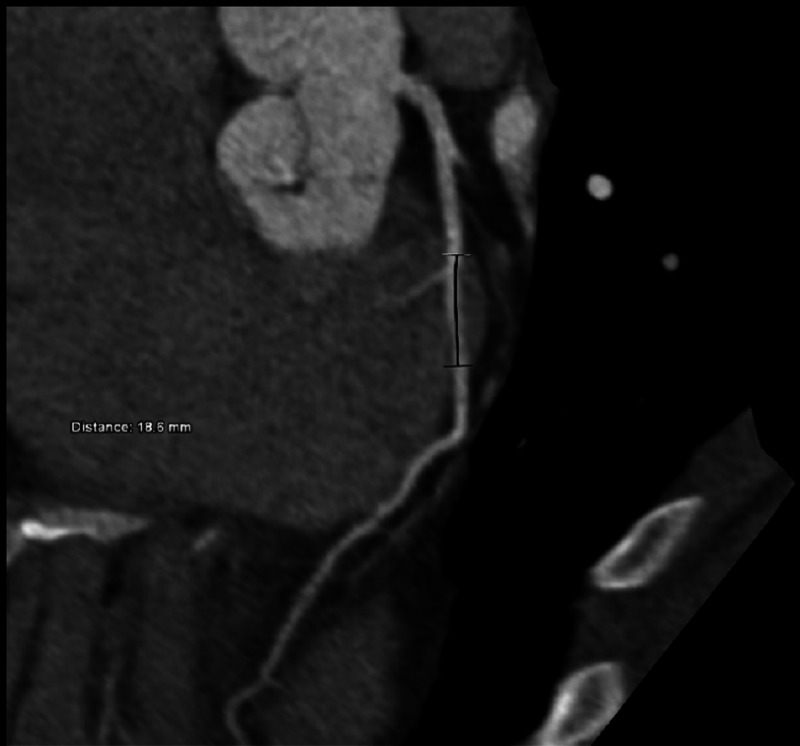
CTA of the patient The image showed mid-LAD deep myocardial bridging, the tunneled segment of about 1.8 cm with about 5 mm depth in the myocardium CTA: computed tomography angiography; LAD: left anterior descending

Although the pain was atypical for cardiac ischemia, we obtained a CT angiography (CTA) because the other investigations could not determine the cause of her chest pain. Interestingly, her CTA revealed a short intramyocardial course of the LAD segment (Figure 2).

The diagnosis of MB was made at that time, and our patient was treated medically with beta-blockers and calcium channel blockers for a few months with follow-up observations. She remained symptomatic, with no significant improvement in her chest pain. We considered surgery to help the patient relieve her symptoms in consultation with the patient, who provided written informed consent once educated on the potential risks and benefits. Surgical myotomy was successful, resulting in the resolution of the patient’s symptoms. Postoperative angiography showed a fractional flow reserve (FFR) of 0.87 in the distal LAD, which confirmed a technically good result.

## Discussion

MB is usually a benign congenital anomaly of the coronary arteries, and most cases are discovered during autopsy [6]. It is more common in older men and, in most cases, involves the LAD (77.2%). MB can also occur in the left circumflex artery (40%) and right coronary artery (36%) [7]. The length of the MB ranges from 5 mm to 70 mm [6].

While MB is usually benign, it can lead to acute coronary syndrome and ventricular and supraventricular arrhythmias, and even ventricular rupture, cardiac arrest, and sudden death in some cases [5,8]. MB is commonly detected on CTA, although only one-third of cases get compressed on systole. Stable angina is the most common presentation. Our patient presented mainly with chest pain that was not suggestive of cardiac etiology, given her pain was stabbing and occurred at rest, combined with her young age and lack of risk factors for cardiac disease.

Coronary angiography, intracoronary Doppler, CTA, positron emission tomography, and echocardiography are the common modalities to detect the significance of MB [6]. For our patient, routine examinations revealed no causes of her pain, and she was significantly symptomatic.

A retrospective review of 31 patients who underwent surgical myotomy for significant MB has reported that all patients became symptom-free following the surgical procedure, with an improvement in the New York Heart Association class from I-III to I-II [9]. Our patient was very symptomatic and was treated medically for approximately six months. Surgical repair of a very deep and long MB carries a high risk, particularly when the right ventricle is punctured intraoperatively. However, the right ventricle hole was easily repaired in our patient, and her postoperative angiography revealed an FFR of 0.87 in the distal LAD, which confirmed a technically good result.

## Conclusions

This report discussed our experience of treating a rare case of mid-LAD bridging, which had caused recurrent atypical chest pain in a young woman. A high index of suspicion of MB should always be maintained when evaluating patients with typical or atypical chest pain given its high prevalence in autopsy findings and dangerous complications such as arrhythmias and sudden death. We believe this case report will prompt our colleagues to consider MB as a potential cause of atypical chest pain when no obvious diagnosis is found.
